# Cerium Oxide Nanoparticles: A Brief Review of Their Synthesis Methods and Biomedical Applications

**DOI:** 10.3390/antiox7080097

**Published:** 2018-07-24

**Authors:** Atul Dhall, William Self

**Affiliations:** 1 Colleges of Nanoscale Science and Engineering, SUNY Polytechnic Institute, Albany, NY 12203, USA; adhall@sunypoly.edu; 2 Burnett School of Biomedical Sciences, College of Medicine, University of Central Florida, Orlando, FL 32816, USA

**Keywords:** cerium oxide nanoparticles, nanoceria, lanthanide, redox-cycling, antioxidants, oxidative stress, green synthesis, biocompatibility, enzyme mimetics, personalized medicine

## Abstract

Cerium oxide nanoparticles (CeNPs) exhibit antioxidant properties both in vitro and in vivo. This is due to the self-regeneration of their surface, which is based on redox-cycling between 3+ and 4+ states for cerium, in response to their immediate environment. Additionally, oxygen vacancies in the lattice structure allow for alternating between CeO_2_ and CeO_2−*x*_ during redox reactions. Research to identify and characterize the biomedical applications of CeNPs has been heavily focused on investigating their use in treating diseases that are characterized by higher levels of reactive oxygen species (ROS). Although the bio-mimetic activities of CeNPs have been extensively studied in vitro, in vivo interactions and associated protein corona formation are not well understood. This review describes: (1) the methods of synthesis for CeNPs, including the recent green synthesis methods that offer enhanced biocompatibility and a need for establishing a reference CeNP material for consistency across studies; (2) their enzyme-mimetic activities, with a focus on their antioxidant activities; and, (3) recent experimental evidence that demonstrates their ROS scavenging abilities and their potential use in personalized medicine.

## 1. Introduction

Cerium, a rare earth metal, is the first element of the lanthanide series in the periodic table. The 4f orbitals of rare earth metals are adequately shielded by 5p and 4d electrons, leading to interesting catalytic properties [1]. Unlike most rare earth metals, cerium can exist in both 3+ and 4+ states [2]. Thus, cerium oxide exists as both CeO_2_ and Ce_2_O_3_ in the bulk state. At the nanoscale, however, cerium oxide has a mix of cerium in the 3+ and 4+ states on the nanoparticle surface. With a decrease in nanoparticle diameter, the number of 3+ sites on the surface increase and oxygen atoms are lost (oxygen vacancies) [3,4]. This is depicted by an overall structure of CeO_2−*x*_.

Cerium oxide nanoparticles (CeNPs, nanoceria) are widely used in chemical mechanical polishing/planarization [5], corrosion protection [6], solar cells [7], fuel oxidation catalysis [8], and automotive exhaust treatment [9]. Pertinent to this review, CeNPs also display many bio-relevant activities-mimicking superoxide dismutase (SOD) [10], catalase [11], peroxidase [12], oxidase [13], and phosphatase [14], and scavenging hydroxyl radicals [15], nitric oxide radicals [16], and peroxynitrite [17].

Reactive Oxygen Species (ROS) are released as by-products in aerobic metabolism and they are routinely linked to oxidative stress (increased levels of intracellular ROS contributing to many diseases). However, ROS primarily act as signaling molecules in physiological processes. For a current and detailed understanding of ROS, readers are referred to an extensive review by Schieber et al. [18] that describes the two faces of ROS—redox signaling and oxidative stress. In this context, antioxidants can be defined as substances that scavenge ROS or inhibit their production. Interest in studying antioxidants grew after a study that described the potential benefits of vitamin E on cardiac health [19]. The activity of metal and metal-based nanoparticle systems and their interactions with ROS depend on their microenvironment. It is well established that metal and metal oxide nanoparticles exhibit antioxidant properties [20]. Since naturally occurring small antioxidant molecules have limited absorption into the body [19], nanoparticles have been investigated as carriers for antioxidant molecules [20]. On the other hand, metal and metal-based nanoparticle systems can also be used for prooxidant treatment strategies [21,22,23,24].

The bio-relevant activities of CeNPs earmark them for use in potential pharmacological agents [25], drug delivery [26,27,28], and bioscaffolding [29,30]. The basis for these activities of CeNPs is the thermodynamic efficiency of redox-cycling between 3+ and 4+ states on their surface [10] and their unique ability to absorb and release oxygen [31]. While it was initially thought that both oxygen vacancies and the redox-cycling between cerium in 3+ and 4+ states are involved in the antioxidant activities of CeNPs [6,10], it is now accepted that redox-cycling is solely responsible for all antioxidant properties [32]. This suggests that the surface ratio of Ce^3+^/Ce^4+^ plays a key role in all of the bio-relevant activities of CeNPs. It is worth noting that CeNPs can also show prooxidant properties at lower pH values [13] and high doses [33], and they are known to exhibit potential toxicity based on their synthesis method, concentration, and exposure time, as detailed in a review by Yokel et al. [34]. As explained by Xu et al. [35] in their review on CeNPs and their applications, cerium is not found in the human body and there are no known clearance mechanisms for it. This implies that exposure to cerium would lead to systemic toxicity. These reasons necessitate careful optimization of synthesis parameters to generate non-toxic CeNPs that have either prooxidant or antioxidant properties that are based on the treatment strategy being used.

The interactions of a nanoparticle system with its microenvironment need to be considered while designing effective nanocarriers. It is important to note that polymeric nanocarriers and smart polymer systems can be used to encapsulate enzymes for drug delivery applications [36,37,38,39,40,41,42] and offer good biocompatibility. Such systems can also offer dual responsive programmable drug release [43]. However, CeNP-based treatment strategies have a unique advantage in that they have a self-regenerative antioxidant capability [44].

In the past decade, there have plenty of studies that demonstrate antibody-directed targeted delivery of antioxidant enzymes, like superoxide dismutase and catalase [45,46,47,48]. To achieve similar goals of targeted delivery, there are studies that have demonstrated the use of functionalizing CeNPs with surface groups and stabilizers so that they can be applied for targeted delivery into the body (reviewed by Nelson et al. [44]). Such functionalization though, needs to be fine-tuned to suit the needs of a targeting strategy and ensure that the CeNPs that are involved can self-regenerate their surface. Clearance from the body also needs to be considered while designing effective CeNP-based treatment strategies. Readers are directed to a review by Walkey et al. [49] for detailed information on targeting strategies and the possible routes of clearance.

This review begins with a brief overview of the methods that are routinely used to synthesize CeNPs. Green synthesis methods are highlighted because of their use of biocompatible stabilizers that may provide non-toxic preparation routes. We then briefly describe known enzyme-mimetic and antioxidant activities of CeNPs. Lastly, recent evidence from both in vitro and in vivo studies is presented to provide the reader with an up-to-date account of the potential biomedical applications of CeNPs.

## 2. Synthesis of Cerium Oxide Nanoparticles

The physicochemical properties of any nanoparticle depend on the method of synthesis. For a bio-relevant nanoparticle, synthesis parameters need to be carefully optimized to select for beneficial physicochemical properties in vivo. Different methods of synthesis result in CeNPs of varying size, morphology, and agglomeration. In general, using a polymer or surfactant during synthesis or a coating post-synthesis results in lowered agglomeration of CeNPs in bio-relevant solutions.

An important consideration while synthesizing nanoparticles for use in vivo is the formation of a protein corona that affects both the uptake and clearance of the nanoparticle. Readers are encouraged to read a review by Lynch et al., on the nanoparticle-protein corona [50]. Common methods used to synthesize CeNPs, including recent green synthesis methods, are listed in Table 1 with a relevant example for each method.

### 2.1. Traditional Synthesis Methods

Numerous methods for the synthesis of CeNPs have been reported. These include solution precipitation [51], hydrothermal [52], solvothermal [53], ball milling [54], thermal decomposition [55], spray pyrolysis [56], thermal hydrolysis [57], and sol-gel methods [58,59,60]. While these methods can help to determine the shape and size, Dowding et al. [14] were the first to report fine-tuned control over the surface ratio of Ce^3+^/Ce^4+^. As expected, many of the traditional methods suffer from low biocompatibility. In general, biocompatible coatings of nanoparticles provide greater stability, longer retention times, and lower toxicity by decreasing non-specific interactions. CeNPs have been functionalized using a variety of coatings–polyacrylic acid [61], polyethylene glycol (PEG) [62], dextran [63], polyethyleneimine [64], cyclodextrin [27], glucose [26], and folic acid [28]. Additionally, CeNPs can also be doped with chelating MRI contrast agents, such as gadolinium, to improve their safety while also displaying antioxidant properties [65]. For a comprehensive review of traditional synthesis methodologies and the associated physicochemical properties, readers are directed to a review by Das et al. [66].

### 2.2. Green Synthesis Methods

Recently, bio-directed CeNP synthesis methods that use natural matrices as stabilizing agents have gained importance because they help alleviate concerns of bio-compatibility. Such green chemistry methods provide safer routes for preparing CeNPs [59,60] and they are potentially useful for pharmaceutical applications [67]. In general, these methods provide low-cost and simpler alternatives to traditional synthesis methods. However, conclusions about the biocompatibility of a given green synthesis method should only be made after assessing the protein corona formation for synthesized CeNPs in biological fluid environments. The effect of the surface ratio of Ce^3+^/Ce^4+^ on the biological properties of CeNPs synthesized via green chemistry methods also needs to be investigated [67].

The main strategies involved in the green synthesis of CeNPs are plant-mediated synthesis, fungus-mediated synthesis, polymer-mediated synthesis, and nutrient-mediated synthesis. Plant-mediated methods (phytosynthesis), where plant extracts act as stabilizing and capping agents, result in relatively large CeNPs [68] that are currently not appropriate for biomedical applications [69]. Fungus-mediated methods (mycosynthesis) resolve this by producing smaller CeNPs [70] that are more stable, have higher water dispersibility, and high fluorescent properties [71]. Natural polymers can also aid in the green synthesis of CeNPs and act as stabilizers [72]. An example is the use of PEG to create dispersible nanopowders in aqueous solutions [73]. Nutrient-mediated synthesis, such as in the use of egg white as a substrate to synthesize CeNPs, are extremely cost-effective [72]. Egg white proteins act as stabilizers that result in controlled isotropic growth of small CeNPs. For further information on the green synthesis methods that are available for CeNPs, readers are directed to a review by Charbgoo et al. [67].

As seen from the table, the sheer breadth of techniques available and the resulting physicochemical properties of synthesized CeNPs warrant the development of a reference CeNP material that has well-characterized properties and can be used to maintain consistency across studies.

## 3. Bio-Relevant Activities of Cerium Oxide Nanoparticles

### 3.1. Enzyme Mimetic Activities

#### 3.1.1. Superoxide Dismutase and Catalase Mimetic Activity

Superoxide radicals are one of the most abundant free radicals produced in mammalian cells and that are produced as a result of normal aerobic metabolism. Superoxide radicals are also signaling molecules as well as a key component in the oxidative response to pathogens. An excess of these radicals is normally eradicated by the enzyme superoxide dismutase (SOD) [74]. CeNPs with high 3+/4+ ratios exhibit higher SOD-mimetic activity in comparison to those with lower 3+/4+ ratios [10].

Catalase is responsible for the degradation of H_2_O_2_, a potentially harmful oxidizing agent [75]. CeNPs with low 3+/4+ ratios exhibit higher catalase-mimetic activity in comparison to those with high 3+/4+ ratios [76]. This is in direct opposition to the trend observed for SOD-mimetic activity. Readers are directed to a review by Celardo et al. [25] for detailed mechanisms of these activities. Since CeNPs have both SOD-mimetic and catalase-mimetic activities, the H_2_O_2_ that is generated in the SOD-mimetic process can enter the catalase-mimetic process to produce H_2_O and O_2_. If coordinated, these activities can make CeNPs excellent antioxidants [35].

#### 3.1.2. Phosphatase Mimetic Activity

Phosphatases are enzymes that remove phosphate groups from their substrates by hydrolysis of esters into phosphate ions [77]. CeNPs with low 3+/4+ ratios exhibit phosphatase-mimetic abilities for both artificial phosphate substrates [78] and bio-relevant substrates, like ATP [14,79] while CeNPs with high 3+/4+ ratios do not display phosphatase-mimetic activity [14,79]. This is the same trend as observed for catalase-mimetic activity. However, we recently demonstrated that the phosphatase-mimetic activity has distinct active sites from those for catalase-mimetic activity [79]. Given the abundance phosphate anions in biological solutions, it is also important to consider that interactions of CeNPs with phosphate anions increase the catalase-mimetic activity while decreasing the SOD-mimetic activity [80,81].

### 3.2. ROS Scavenging Activities

#### 3.2.1. Scavenging Hydroxyl Radicals

The hydroxyl radical is one of the most biologically active free radicals [82]. CeNPs have been shown to partly eliminate hydroxyl radicals in a size-dependent manner [83]. One of the earliest studies of this behavior, conducted by Das et al. [15], demonstrated that CeNPs with mixed valence states have a neuroprotective effect on adult rat spinal cord neurons.

#### 3.2.2. Scavenging Nitric Oxide Radicals and Peroxynitrite

The nitric oxide radical is a gaseous free radical with both beneficial and damaging biological effects [84]. Similar to their catalase-mimetic activity, CeNPs with low 3+/4+ ratios exhibit higher scavenging of nitric oxide radicals [16]. Nitric oxide radicals can react with superoxide radicals to make peroxynitrite. CeNPs have also been shown to accelerate the decay of peroxynitrite [17]. Since CeNPs interact with both peroxynitrite and its breakdown products, their scavenging of peroxynitrite is independent of the 3+/4+ ratio.

## 4. Evidence for Bio-Relevant Activities of Cerium Oxide Nanoparticles

This section provides the reader with recent evidence of CeNPs’ antioxidant activities. Each synopsis lists the synthesis method that is used as it is important for researchers to keep these methods in mind before utilizing similar strategies in new experiments. A list of recent literature detailing the biomedical use of CeNPs is shown in Table 2. The sub-sections that follow provide details on each of these studies. Readers are referred to a comprehensive review by Das et al. [66] for similar evidence prior to 2013.

### 4.1. In Vitro Studies

Pezzini et al. [85] demonstrated the protective effects of CeNPs on primary human skin fibroblasts that were exposed to a pro-oxidative insult. Monodispersed CeNPs with an average diameter of 2 nm were purchased from Alfa Aesar. Fibroblast proliferation was assessed after 24 and 72 h of incubation with CeNPs at concentrations of 0, 100, and 200 µg/mL. It was found that CeNPs were internalized, displayed strong ROS scavenging activity, and did not affect the viability of the fibroblasts. Additionally, CeNPs affected mitochondrial function by leading to an increase in ATP production, but preserved mitochondrial membrane potential. This study serves as a proof-of-principle of the applications of CeNPs for a wide range of conditions that are associated with the accumulation of oxidative stress and alteration of mitochondrial metabolism.

Zeng et al. [86] demonstrated that PEGylated-CeNPs can mitigate pro-inflammatory M1-polarization and advance anti-inflammatory M2-polarization by scavenging ROS triggered by stress stimuli in microglial BV-2 and PC-12 cells. Near-spherical CeNPs were synthesized, as described by Lee et al. [95]. PEGylated-CeNP treated microglia showed neuroprotective effects on the co-cultured neurons after Oxygen and Glucose Deprivation/Reoxygenation stimulus by blocking the pro-inflammatory NF-κB pathway. This study provides a new strategy for reshaping the immune-microenvironment using the phenotypic activation of microglia.

Sack et al. [87] examined the use of redox-active CeNPs in combination with a conventional therapeutic, doxorubicin, for cancer therapy. The CeNPs that were used in this study were synthesized as described by Karakoti et al. [96]. Antitumor activity of doxorubicin is primarily mediated by DNA damage, cell-cycle arrest, and apoptosis. While doxorubicin is effective against cancer cells, it also affects healthy cells [97]. The authors compared the antitumor activity of CeNPs with doxorubicin and demonstrated that CeNPs enhanced the antitumor activity of doxorubicin in human melanoma cells (A375 cells), in context of cytotoxicity, ROS formation, and oxidative damage. While both CeNPs and doxorubicin exerted cytotoxic effects on A375 cells, when used together, the viability of A375 cells decreased further than with each agent alone. Additionally, CeNPs abolished the toxic effects of doxorubicin on human dermal fibroblasts (HDFs). Such approaches involving the supplementation of conventional chemotherapeutics with CeNPs may offer novel strategies in cancer therapy. The same group has also investigated the potential use of CeNPs in brain tumor-related treatment. Sack-Zschauer et al. [98] demonstrated that CeNPs killed malignant glioma cells while protecting healthy cells. CeNPs (water-based suspension of 1.5 mg/mL and 1–10 nm) were purchased from Sciventions and stabilized in sodium polyacrylate (1.27 mg/mL). The authors reported that CeNPs had a cytotoxic effect on anaplastic astrocytoma (grade III glioma) cells while displaying no cytotoxicity towards microvascular endothelial cells.

Patel et al. [88] demonstrated that human monocyte leukemia cells (THP-1 cells) can be used as a model to evaluate the uptake and free radical scavenging ability of CeNPs. CeNPs were synthesized using slight modifications to a basic strategy that was described by Hirst et al. [99]. Internalization of CeNPs was shown to increase in a concentration-dependent manner in THP-1 cells between 10 and 100 µg/mL. Additionally, CeNPs reduced the amount of ROS without exhibiting cytotoxicity. Thus, unlike other oxide nanoparticles, which induce ROS generation in the cytoplasm [100], CeNPs retained their antioxidant activity in the cytoplasm after rapid internalization by THP-1 cells.

Sadhu et al. [89] demonstrated that high CeNP concentrations in tobacco BY-2 cells induce cytotoxicity and impair metabolic activity while low concentrations exhibit antioxidant activity. CeNPs that were used in this study (under 25 nm) were purchased from Sigma-Aldrich Chemical Co., (Bengaluru, India). The authors treated tobacco BY-2 cells with CeNP concentrations of 10, 50, and 250 µg/mL for 24 h. While concentration-dependent accumulation of Ca^2+^ and ROS was observed for all CeNPs sets, significant DNA damage and alterations in antioxidant defense systems were observed for higher CeNP concentrations (50 and 250 µg/mL). Further, CeNPs at 10 µg/mL did not induce genotoxicity and led to reduced DNA breakage in cells tha were exposed to H_2_O_2_. Their results point to an alternative autophagy-mediated, antioxidant, and geno-protective role of CeNPs.

### 4.2. In Vivo Studies

Kwon et al. [90] reported the design and synthesis of triphenylphosphonium-conjugated CeNPs that localized to mitochondria and suppressed neuronal death in 5XFAD transgenic mice (Alzheimer’s disease model). CeNPs were synthesized using hydrolytic sol-gel reactions. Mitochondrial dysfunction can lead to abnormal levels of ROS and subsequently cause neuronal cell death. Targeting CeNPs to mitochondria is a promising therapeutic approach for neurodegenerative diseases. The authors synthesized small and positively charged triphenylphosphonium-conjugated CeNPs that are capable of localizing to mitochondria in various cell lines and mitigating reactive gliosis while suppressing neuronal death. This can serve as a novel strategy in the development of mitochondrial therapeutics for Alzheimer’s disease and other neurodegenerative diseases.

Hijaz et al. [91] demonstrated that CeNPs significantly reduced tumor growth in an ovarian cancer xenograft nude model. A synthesis strategy that is based on methods used by Das et al. [101] and Cimini et al. [102] was used to make CeNPs and folic acid-CeNPs, respectively. A2780 generated mouse xenografts were treated with 0.1 mg/kg CeNPs, 0.1 mg/kg folic acid-CeNPs, and 4 mg/kg cisplatinum by intra-peritoneal injections. Mice that were treated with folic acid-CeNPs had a lower tumor burden when compared to those treated with just CeNPs. Combining folic acid-CeNPs with cisplatinum further decreased the tumor burden. Additionally, folic acid-CeNPs reduced vimentin expression, thus indicating a potential ability to limit ovarian tumor metastasis.

Wong et al. [92] used the P23H-1 rat (autosomal dominant retinitis pigmentosa model) to understand the cellular mechanisms and duration of the CeNPs’ catalytic activity in preventing photoreceptor cell loss. CeNPs were prepared using wet chemistry methods, as described by Karakoti et al. [103]. An increase in rod and cone cell function post-injection was observed. Additionally, CeNP treatment led to a reduction in apoptotic cells and lipid peroxidation in the retinas. This study adds to the list of rodent retinal disease models that demonstrate a delay in disease progression with just one application of CeNPs.

Rocca et al. [93] investigated the antioxidant effects of CeNPs as an approach for obesity treatment in Wistar rats. CeNPs interfered with the adipogenic pathway and hindered triglyceride accumulation. CeNPs were purchased from Sigma and it had characteristics that were similar to those used in a study by Ciofani et al. [104]. They were administrated intraperitoneally twice a week for six weeks at a dose of 0.5 mg/kg in 500 μL of sterile water. Transcriptional analysis following in vivo treatment revealed a down-regulation of *Lep*, *Bmp2*, *Twist1*, *Angpt2*, and *Ddit3*, and an up-regulation of *Irs1* and *Klf4* expression. Overall, CeNPs contributed to a reduction in weight gain and lowered the plasma levels of insulin, leptin, glucose, and triglycerides.

Manne et al. [94] investigated the use of CeNPs in the prophylactic treatment of hepatic ischemia reperfusion injury in Sprague Dawley rats. CeNPs were obtained from U.S. Research Nanomaterials and are characterized as in Manne et al. [105]. Partial hepatic ischemia was induced for 1 h in the left lateral and median lobes. This was followed by 6 h of reperfusion. Prophylactic treatment with CeNPs (at 0.5mg/kg) led to a decrease in alanine aminotransaminase, lactate dehydrogenase, hepatocyte necrosis, macrophage derived chemokine, macrophage inflammatory protein-2, keratinocyte chemoattractant (KC)/human growth-regulated oncogene (GRO), myoglobin, and plasminogen activator inhibitor-1.

## 5. Conclusions and Future Perspectives

Oxidative stress is implicated in the development and progression of many diseases. The broad range of CeNPs’ antioxidant activity and their ability to self-regenerate their surface makes them strong candidates for use as in vivo ROS scavengers. However, to be considered as potential therapeutic agents, it is necessary to optimize their synthesis methods, surface chemistry, and concentration to select the beneficial physicochemical properties. For consistency, such endeavors must begin by establishing CeNP reference materials for pertinent disease models. Eventually, all toxicological concerns also need to be addressed.

It is important to note that the physicochemical properties of CeNPs reported in vitro differ from those under physiological conditions. In particular, the natural protein corona associated with CeNPs in vivo is primed to be the focus of studies that describe existing/identify new biomedical applications. Any pharmacokinetic improvement will involve CeNPs with biocompatible coatings and effective targeting strategies.

Green synthesis methods that use biocompatible stabilizers are increasingly gaining relevance in the production of CeNPs and their biomedical applications. Undoubtedly, interdisciplinary studies that are based in material science and tailored towards identifying new biomedical applications of CeNPs will continue to clarify their physicochemical properties and catalytic activities.

## Figures and Tables

**Table 1:** Common methods used to synthesize cerium oxide nanoparticles.

Method of Preparation		Particle Size (nm)	Morphology	Reference
Traditional approaches	Precipitation	15	Spherical	[51]
	Hydrothermal	5	Octahedral	[52]
	Solvothermal	~8	Polyhedral	[53]
	Spray Pyrolysis	~17	Cubic	[56]
Green approaches	Plant-mediated	36	Spherical	[68]
	Fungus-mediated	5	Spherical	[70]
	Polymer-mediated	~2	Spherical	[73]
	Nutrient-mediated	25	Spherical	[72]

**Table 2:** Recent literature on the biomedical studies involving cerium oxide nanoparticles (CeNPs).

Type of Testing	Cell Line/Animal Model	Use	Reference
In Vitro	Primary human skin fibroblasts	Alteration of mitochondrial metabolism	[85]
	BV-2 and PC-12	Phenotypic activation of microglia to reshape immune microenvironment	[86]
	A375	Combinational cancer therapy with doxorubicin	[87]
	THP-1	Evaluating uptake and ROS scavenging ability of CeNPs	[88]
	BY-2	Autophagy-mediated antioxidant and geno-protective role of CeNPs	[89]
In Vivo	5XFAD mice	Suppressing neuronal death in Alzheimer’s disease	[90]
	Nude Mice	Reduction of tumor growth in ovarian cancer	[91]
	P23H-1 rat	Preventing photoreceptor cell loss in autosomal dominant retinitis pigmentosa	[92]
	Wistar rat	Obesity treatment	[93]
	Sprague Dawley rat	Prophylactic treatment of hepatic ischemia reperfusion injury	[94]
